# Effect of Surfactant Type and Sonication Energy on the Electrical Conductivity Properties of Nanocellulose-CNT Nanocomposite Films

**DOI:** 10.3390/ijms19061819

**Published:** 2018-06-20

**Authors:** Sanna Siljander, Pasi Keinänen, Anna Räty, Karthik Ram Ramakrishnan, Sampo Tuukkanen, Vesa Kunnari, Ali Harlin, Jyrki Vuorinen, Mikko Kanerva

**Affiliations:** 1 Laboratory of Materials Science, Tampere University of Technology, FI-33720 Tampere, Finland; pasi.keinanen@tut.fi (P.K.); anna.raty@tut.fi (A.R.); karthik.ramakrishnan@tut.fi (K.R.R.); jyrki.vuorinen@tut.fi (J.V.); mikko.kanerva@tut.fi (M.K.); 2 BioMediTech, Tampere University of Technology, FI-33720 Tampere, Finland; sampo.tuukkanen@tut.fi; 3 VTT Research Center, FI-02044 Espoo, Finland; vesa.kunnari@vtt.fi (V.K.); ali.harlin@vtt.fi (A.H.)

**Keywords:** nanocellulose, carbon nanotubes, nanocomposite, conductivity, surfactant

## Abstract

We present a detailed study on the influence of sonication energy and surfactant type on the electrical conductivity of nanocellulose-carbon nanotube (NFC-CNT) nanocomposite films. The study was made using a minimum amount of processing steps, chemicals and materials, to optimize the conductivity properties of free-standing flexible nanocomposite films. In general, the NFC-CNT film preparation process is sensitive concerning the dispersing phase of CNTs into a solution with NFC. In our study, we used sonication to carry out the dispersing phase of processing in the presence of surfactant. In the final phase, the films were prepared from the dispersion using centrifugal cast molding. The solid films were analyzed regarding their electrical conductivity using a four-probe measuring technique. We also characterized how conductivity properties were enhanced when surfactant was removed from nanocomposite films; to our knowledge this has not been reported previously. The results of our study indicated that the optimization of the surfactant type clearly affected the formation of freestanding films. The effect of sonication energy was significant in terms of conductivity. Using a relatively low 16 wt. % concentration of multiwall carbon nanotubes we achieved the highest conductivity value of 8.4 S/cm for nanocellulose-CNT films ever published in the current literature. This was achieved by optimizing the surfactant type and sonication energy per dry mass. Additionally, to further increase the conductivity, we defined a preparation step to remove the used surfactant from the final nanocomposite structure.

## 1. Introduction

Conductive composite materials with micrometer and nanoscale fillers, like metallic powders, carbon black, graphite and carbon fibers, are used in many applications, such as antistatic films and electromagnetic interference (EMI) shielding. Electrical conductivity of 0.01 S/cm or higher is required for the composite to be considered conductive, while materials with lower conductivity can be used as antistatic and semiconducting materials. One of the drawbacks with most fillers is that the filler content ratio needs to be as high as 50 wt. % to achieve the percolation threshold (i.e., the critical concentration of filler that corresponds to the sharp rise of conductivity). However, this high filler content ratio might lead to a decrease in the resultant composite’s mechanical properties [1,2]. Nanomaterials, such as carbon nanotubes (CNTs) and graphene, play a role in the development of future composite materials. For example, CNTs and graphene have been used to toughen matrix polymers [3], to adjust barrier properties of nanocomposite films [4], and to form hierarchical reinforcements [5]. It is possible to attain the percolation threshold in the insulating polymer matrix at a low CNT concentration due to their excellent electrical, mechanical and thermal properties.

Individual CNTs are part of a group of the strongest and most conductive nanomaterials known [6]. Additionally, CNTs can carry higher current density than any other known material, with its highest measured value being 109 A/cm^2^ [7,8]. However, to obtain an ideal conductive network, the carbon nanotubes have to be well separated and homogenous dispersion should be maintained in the final product. Without efficient dispersion, filler aggregates act as defect sites, which leads to lower mechanical performance [9,10]. As the most abundant polymer on earth, cellulose is a promising and well-known material that can be used as a matrix in nanocomposites.

Cellulose is environmentally conscious, low-cost, strong, dimension-stable, non-melting, non-toxic and is a non-metal matrix. The interest towards nanoscale cellulose has increased during the past few years because of its inherent properties, including its good mechanical properties, which are better than those of the respective source biomass material [11]. Cellulose-based micro-/nanofibrils (MFC/NFC) can be extracted from various types of plant fibers using mechanical forces, chemical treatments, enzymes or combinations of these. The most typical approach, however, is to apply wood pulp and mechanical methods such as homogenization, microfluidization, microgrinding and cryocrushing. Finally, after fibrillation, the width of NFC is typically between 5 and 20 nm, with a length of several micrometers. Nanocellulose (NFC) has hydroxyl groups in its structure and is therefore associated with high aspect ratio and strong hydrogen bonds formed between nanocellulose fibers [12]. These bonds enhance mechanical properties and enable the formation of free standing films. A combination of CNTs and cellulose I provides a conductive nanocomposite network. CNT-cellulose composites have been reported to be used as supercapacitor electrodes [13,14], electromagnetic interference shielding devices [15], chemical vapor sensors [16], water sensors [17,18], and pressure sensors [19].

There are different manufacturing methods for the fabrication of CNT-cellulose nanocomposites, but all the methods typically include (1) a phase of dispersing CNTs into a solution, and (2) an impregnation phase into the cellulose substrates (e.g., paper, filter paper) [15,16,20,21,22,23]. Alternatively, the dispersion can be used as a wet component with bacterial cellulose [24,25], with cellulose I and regenerated cellulose fibers [13,18,26] or in an aerogel form [17]. The processing of nanocellulose in an aqueous medium is the most common way due to its tendency to react with water, and strong affinity to itself and hydroxyl group containing materials [12]. Chen et al. [27] showed that NFCs and CNTs can form a three-dimensional conductive network structure in a gel-film morphology to achieve high electrical conductivity.

The properties of the nanocellulose-CNT composites are affected by the quality of CNT dispersion, amount of structural and oxidative defects in the graphitic structure of the CNTs, the aspect ratio of the CNTs after the disaggregate treatment, the strength of the matrix, and the interactions between the CNTs and the cellulose matrix. [28] The key challenge in numerous industrial applications is to achieve uniform and stable CNT dispersion. The homogenization phase is vital to maximize the excellent mechanical, electrical and thermal properties of the CNTs and the eco-friendly, strong and low-cost nanocellulose matrix. This is particularly important in the case of submicron- or nanometer-sized particles. In these scales, the surface chemistry plays an important role, managing the particle dispersion within the final product [29]. CNT dispersions are challenging because as the surface area of particles increases, the attractive forces between the aggregates [29] and the high aspect ratio enable the entanglement and bundling of CNTs [30]. There are two phenomena that affect CNT dispersions: nanotube morphology and the forces between the tubes. Entanglement of CNTs occurs due to tube morphology, as well as molecular forces, high aspect ratio, and high flexibility. Dispersing these entangled aggregates is difficult without damaging the nanotubes. Both CNT and aggregate size are expected to play a crucial role in the achieved level of electrical conductivity [31].

Two typical dispersion methods for CNTs include high shear mixing and pure sonication [13,15,16,19,20,21,24,32]. Sonication is based on ultrasonic waves that generate microscopic bubbles or inertial cavitation, which produces a shearing action. This results in liquid and suspended particles becoming intensely agitated. Another common technique is to use a centrifuge in one of the processing steps to extract the unwanted agglomerates from the supernatant, but this additional phase takes time and effort and affects the concentration of dispersed particles in the dispersion. In general, sonication is superior to shear mixing, especially for low-viscosity systems [33], where conventional mixing does not create high enough strain rates to disintegrate the CNT aggregates.

Another issue in the manufacturing of films using NFC is the shrinkage and distortion of the structure because of faster evaporation rate on surface than the mass transport of moisture within the material. When strong enough gradient occurs, film distortions emerge because of local stresses [34,35].

One widely used method for CNT dispersion is the non-covalent method. In this method, chemical moieties are adsorbed onto the surface of CNTs, the CNTs are non-covalently dispersed in a water medium, and the resultant mixture is sonicated in the presence of the moieties, namely surfactants. Surfactants are a group of organic compounds that have a hydrophilic head and a hydrophobic tail, and they are commonly used as detergents, wetting agents, emulsifiers, foaming agents and dispersants. The advantage of the non-covalent method lies in the fact that it does not deteriorate the electronic structure of the CNTs’ graphitic shells, maintaining their high electrical conductivity. Good dispersion can be achieved by having a mixture of both nanocellulose and carbon nanotubes with the help of surfactants, as the surfactants lower the interfacial free energy between the particles. Table 1 lists information about surfactants and their properties used in this study.

In the current literature, there are several different types of surfactants used for dispersing nanocellulose and carbon nanotubes. Choosing a surfactant type for effective dispersion of nanotubes through surfactant adsorption is complicated, as the results in the published literature often give contradictory results. For example, some researchers [29] have suggested that ionic surfactants are preferable for creating aqueous dispersions. However, the non-ionic surfactant Triton X-100 was shown to be a better surfactant than the anionic surfactant SDS, which was attributed to the π-π stacking ability of the former. The quality of the NFC-CNT dispersion is dependent on the nature of the surfactant, the concentration and the type of interactions between the surfactant and dispersing particles [36]. It has been stated that, for dispersing CNTs it is preferable for the surfactant to have a relatively high HLB (hydrophilic-lipophilic balance) value [29]. This assumption was proven false in our previous study [37]. Not only are the surfactant’s nature and energy carried into the dispersed system, but the concentration of the surfactant also has a crucial role in the dispersion process [38]. Too high a surfactant concentration may negatively affect conductivity properties by blocking off the charge transport through the CNT network [39]. In addition, a low surfactant concentration can cause re-aggregation, because a sufficient amount is required to cover CNT surfaces to prevent re-aggregation [39,40]. It has been shown that an efficient CNT dispersion is only possible when the surfactant concentration is above the critical micelle concentration (CMC) value [41,42,43,44]. In some cases, the surfactant concentration is reported to be higher than the (CMC), but no micelle structures are observed in the dispersion. Presumably, most of the surfactant has been adsorbed onto the surface of the CNTs [40]. In other cases, surfactants can prefer surfactant-surfactant interactions over spreading on the CNT surface [45]. It has also been reported that dispersing agents can form stable dispersions below and equal to their CMC limit [46,47,48,49]. Moreover, it has been noted that commonly, the best results can be reached with a concentration of 0.5 CMC and that any further increase in the concentration of surfactant has only a minor effect [48].

The ISO 14887:2000(E) standard can be used to determine prospective dispersing agents for both cellulose and carbon. We can categorize nanocellulose and CNTs as solids. In that case, when using water as liquid, the category of suitable dispersing agent would be a poly ethylene-oxide (PEO)/alcohol for CNT and PEO/poly propylene oxide (PPO) copolymer for nanocellulose. The standard also provides information about commercial surfactants that fall into the mentioned categories. PEO/PPO copolymer is a suitable surfactant for nanocellulose. The standard denotes that a commercial equivalent is Pluronic^®^. In the case of CNTs, one example of alkyl phenoxy PEO ethanol dispersing agent is Triton™.

The typical approach to the manufacturing of conductive cellulose-CNT films has been to increase CNT weight percentage without optimizing the dispersion procedure or the used surfactants. Also, the effect of the particular ratios of the cellulose, CNT and surfactant toward each other has not been fully investigated. Even though ultrasonication is widely used for the dispersion and stabilization of CNTs, there is not a standard procedure for the sonication process, and different research groups have applied different sonication treatments to their samples. Sonication can cause chemical functionalization but it can also cause defects and breakage of CNTs [1,50,51,52]. This will further affect the performance of CNT-based materials and their applications. It has been found in the current literature that sonication parameters such as sonicator type, sonication time and temperature control vary significantly, with reported sonication times ranging from 2 min with tip sonication to 20 h for bath sonication. Dassios et al. [53] attempted to optimize the sonication parameters for the dispersion of MWCNTs in an aqueous solution. Two critical questions concerning the homogeneity of aqueous suspensions of carbon nanotubes by ultrasonic processing were identified; namely, the dependence of dispersion quality on the duration and intensity of sonication and the identification of the appropriate conditions for retaining the highly desirable initial aspect ratio of the free-standing tubes in the dispersed state. Fuge et al. [54] studied the effect of different ultrasonication parameters (time, amplitude) on undoped and nitrogen-doped MWCNTs in aqueous dispersions and found a nearly linear decrease of the arithmetic mean average in MWCNT length with increasing ultrasonication time.

The aim of this study was to optimize the conductivity of NFC-CNT nanocomposite films using a minimum amount of processing steps (e.g., without centrifugal processing of dispersion or pressing of the film), materials and chemicals. In this paper, NFC and multiwall carbon nanotubes (MWCNT) were used to prepare composite films and study the effect of the sonication energy and surfactant type on the electrical conductivity of the nanocomposite. In addition, we investigated the removal of the surfactant from the nanocomposites and the subsequent effect on the electrical conductivity. To our knowledge this is a novel approach and has not been reported previously. The conductivity properties of the nanocomposites were studied as a function of the used sonication energy amount, as well as with and without the presence of surfactant.

## 2. Results

The impact of sonication energy on electrical conductivity was one of the processing parameters with the highest interest in this study. This was due to the lack of previous research in the current literature. Also, our results show that the surfactant type and sonication energy play a major role in achieving excellent conductivity. In addition to the previously mentioned parameters, removal of surfactant can enhance conductivity values toward levels never seen or reported.

Overall, the shelf-life of the sonicated dispersion samples was significantly long, since samples remained unchanged before the film preparation. Also, sedimentation was not detected, based on the fact that conductivity values were at the same level when measured from both sides of the nanocomposite films. The appearance of the sonicated dispersion samples was identical; however, the consistency and visually inspected viscosity varied with increasing sonication energy. This was observed with Triton X-100 and Pluronic F-127 samples but not in cetyl trimethylammonium bromide (CTAB) surfactant-containing dispersions.

### 2.1. Conductivity of NFC-CNT Nanocomposite Films

Electrical conductivity of the even and uniform centrifugally cast films was measured using the four-probe measuring technique. With this method, it is possible to minimize the contact resistances and thus provide more accurate conductivity measurements than for the commonly used two-terminal measurement. The sheet resistances of prepared and cut nanocomposite films (size 30 mm × 30 mm) were measured using a four-point probe setup made in-house and a multimeter (Keithley 2002, Tektronix, Inc., Beaverton, OR, USA) in four-wire mode. The probes were placed in line, with equal 3 mm spacing. The four-probe setup is described elsewhere in detail [55]. The conductivity measurements were carried out using a 1 mA current and voltage was measured. Measurements were taken before and after removal of surfactant.

**Figure 1:**
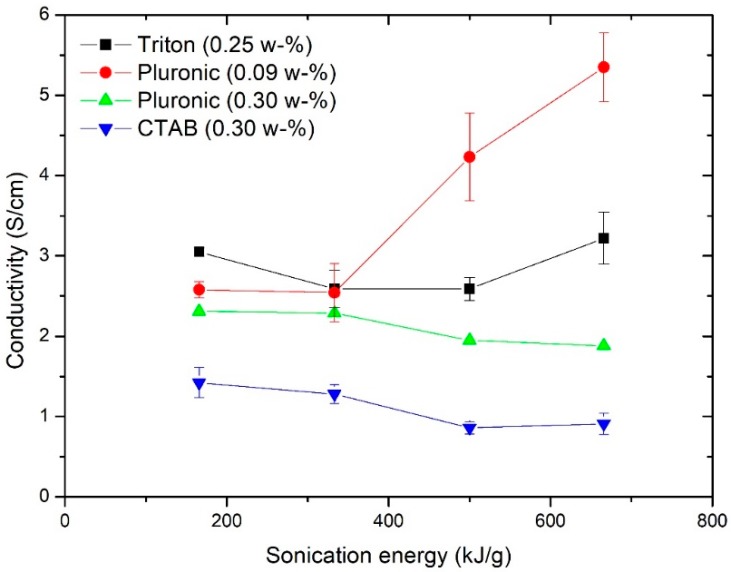
Conductivity of nanocomposite films processed using surfactants Triton X-100, Pluronic F-127 and cetyl trimethylammonium bromide. Concentration in weight percentages.

The selection of the most functional surfactant was an important aspect in this study. This selection was determined based on the sheet resistance measurements. The effect of different surfactants and sonication energy on conductivity is shown in Figure 1. From the conductivity diagrams, the effect of surfactant type can be visually observed and estimated.

According to the standard, our assumption was that non-ionic surfactants would be the most promising surfactants. This was clearly the case, since the films made with surfactants Triton X-100 and Pluronic F-127 outperformed the films made with ionic surfactant CTAB.

Visual observations made with CTAB aqueous dispersion samples after sonication indicated that these samples did not gelate even with a higher amount of sonication energy per dry mass (666 kJ/g). This suggests that the dispersion process may not have been entirely successful, since samples had different consistencies and visually separate particles. The ionic surfactant (CTAB) was used to manufacture films at a 1 to 1 ratio of dry mass content of NFC and CNT. The conductivity of films processed using CTAB decreased as the sonication increased from almost 1.5 S/cm to less than 0.90 S/cm. The conductivity diagram of these films was different in its nature; the highest values were measured with the lowest amount of sonication energy.

Based on the standard Pluronic F-127, surfactant should be compatible with cellulosic materials. The first set of Pluronic F-127 nanocomposite films were done with a 1 to 1 ratio to dry mass content (0.30 wt. %). Results show that conductivity is decreasing as a function of sonication energy. Based on this finding, another set of films was manufactured using a surfactant concentration below the CMC limit (0.09 wt. %). Conductivity results for this set of samples show higher conductivity values than films manufactured using a surfactant concentration higher than the CMC value (0.30 wt. %). Using Pluronic F-127 surfactant, the highest conductivity for nanocomposite films was achieved using a sonication energy of 666 kJ/g. When comparing values of films below and above CMC value the difference is sensational 5.36 S/cm (0.09 wt. %) versus 1.88 S/cm (0.30 wt. %).

For Triton, the highest conductivity value of 3.37 S/cm was achieved with 666 kJ/g sonication energy. It should be noted that almost the same conductivity result (3.02 S/cm) was achieved using just 166 kJ/g of sonication energy.

### 2.2. Effect of Surfactant Removal

**Figure 2:**
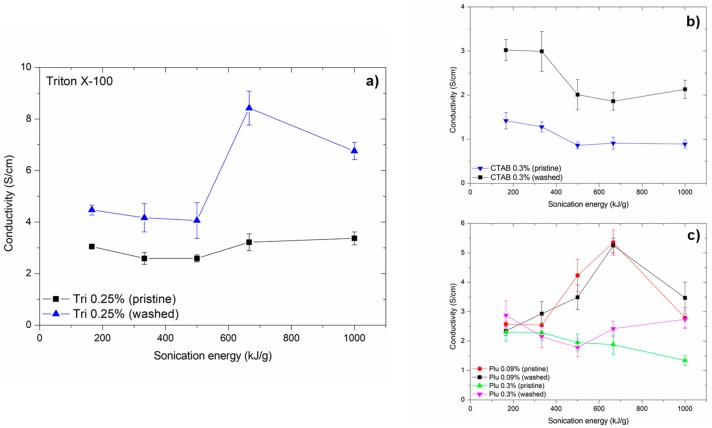
Conductivity of nanocomposite films before and after (**a**) Triton X-100 surfactant removal, (**b**) CTAB surfactant removal, and (**c**) Pluronic surfactant removal.

Conductivity measurements were also carried out after the removal of the surfactant used in the dispersing phase. Triton X-100 and Pluronic F-127 films were acetone treated and CTAB films were treated with ethanol. It can be clearly seen in Figure 2 that removal of surfactant has a strong effect. Removal of surfactant from films made with CTAB increased the conductivity significantly; the maximum conductivity was 3.02 S/cm for 166 kJ/g sonication energy. However, the films expressed a decrease in conductivity at sonication energy similar to the films with the surfactants present. For Pluronic F-127 films (Figure 2c) made below the CMC limit of the surfactant, the removal of surfactant did not have a significant effect on the conductivity. Even though there was no clear trend, the film with surfactant had somewhat higher conductivity than the one where surfactant was not present. For Pluronic F-127 (0.30 wt. %), the shape of the diagram differed from other previous sets. The films initially exhibited a decrease in conductivity as a function of increasing sonication energy, and the highest values were measured for the samples sonicated at the least energy, but also for the highest amount of sonication energy. For the samples with high conductivity, the removal of the non-ionic surfactant increased the conductivity. The highest value obtained from these measurements was 2.88 S/cm for a sonication energy of 166 kJ/g. A dramatic increase was observed in conductivity values of films manufactured with surfactant Triton X-100 (Figure 2a): the conductivity increases from approximately 3.0 S/cm to a value of 8.42 S/cm when the non-ionic surfactant was removed (sample was sonicated at 666 kJ/g). It should be noted that the films containing the surfactant did not exhibit as strong a sensitivity to increasing sonication energy (as those without surfactant).

It is well known that surfactants can plasticize the structure of composites and interfere with conductivity properties by situating themselves at the interface between the conductive particles and matrix. This phenomenon was demonstrated when the properties of the nanocomposite films were compared in this study. Firstly, there was a clear increase in the conductivity of the films processed using the Triton X-100 and CTAB surfactants due to the removal of the surfactant. The diagrams (pristine vs. washed) were similar in their trend, and a clear increase in terms of conductivity was observed. When using the surfactant Pluronic F-127 for processing, a clear conclusion could not be made because the conductivity diagrams did not show a corresponding, monotonic trend due to surfactant removal. However, the results showed that, when the surfactant is present in the film structure, the effect of interference by Pluronic F-127 (concentration below CMC) on the electrical conductivity is at its minimum.

### 2.3. Comparison to Previous Results

**Figure 3:**
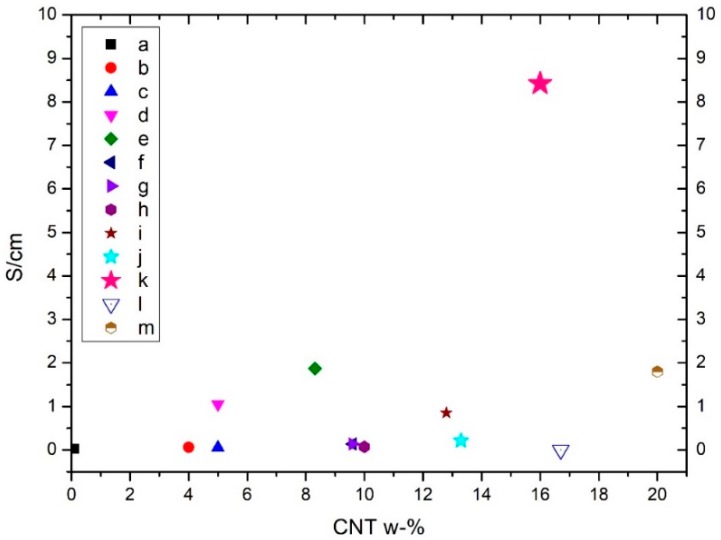
Comparison of obtained electrical conductivity of NFC-CNT nanocomposite films from the current literature. Pink star (letter k) refers to our data (Triton X-100), while other letters refer to a [9], b [56], c [57], d [58], e [22], f [9], g [24], h [57], i [59], j [15], l [32] and m [27].

When comparing our nanocomposite film’s conductivity results to previous studies, we found that our results were superior to reported values. In Figure 3 are illustrated electrical conductivity results from studies that have used native NFC and manufactured homogenous nanocomposites from it. For non-ionic surfactants, the highest conductivity value found was 0.022 S/cm at a 10 wt. % CNT loading [17]. In our study, the highest value was 8.42 S/cm after removing Triton X-100 and, likewise, 5.35 S/cm with Pluronic F-127 still present in the film.

Huang et al. [57] reported the results of a multiphase process which was used to accomplish a conductivity of 0.072 S/cm using MWCNT-doping at 10 wt. % and 0.056 S/cm with 5 wt. % doping with cotton linters and CTAB as a surfactant. CTAB surfactant was also used with bacterial nanocellulose and CNTs, where the conductivity was 0.027 S/cm (MWCNT 0.1 wt. %) [9]. Also, Yoon et al. [24] used bacterial cellulose as a matrix and obtained conductivity of 0.14 S/cm with 9.6 wt. % MWCNT loading. Electrical conductivity of TEMPO-oxidized cellulose films with 16.7 wt. % concentration of MWCNTs was 0.001 S/cm, which is lower than the conductive material limit [32]. For chitosan-cellulose-CNT membranes, Xiao et al. [56] accomplished conductivity of 0.062 S/cm with a 4 wt. % content of MWCNTs. By using comparable materials, but by applying a filtering method, Yamakawa et al. [58] obtained a 1.05 S/cm electrical conductivity with a 5 wt. % MWCNT loading and Chen et al. 1.8 S/cm with 20 wt. % MWCNT. They were able to increase the conductivity to a value of 5.02 S/cm using a chemical alkali treatment.

In addition, studies about manufacturing conductive cellulose composites via coating cellulosic filter paper with a CNT dispersion have revealed rather good results, but the consistency of the materials is not homogeneous—not exactly an integral composite. Lee et al. [15] achieved conductivity of 1.11 S/cm using 13.3 wt. % MWCNT. Mondal et al. [59] reported conductivity values after using a dipping method, and they reached 0.85 S/cm with a 12.8 wt. % carbon nanofiber (CNF) content. Fugetsu et al. [22] manufactured conductive cellulose-based composites using a traditional paper making process with 8.32 wt. % CNT concentration and, finally, a conductivity of 1.87 S/cm was obtained.

### 2.4. Characterization of Nanocomposite Structure

**Figure 4:**
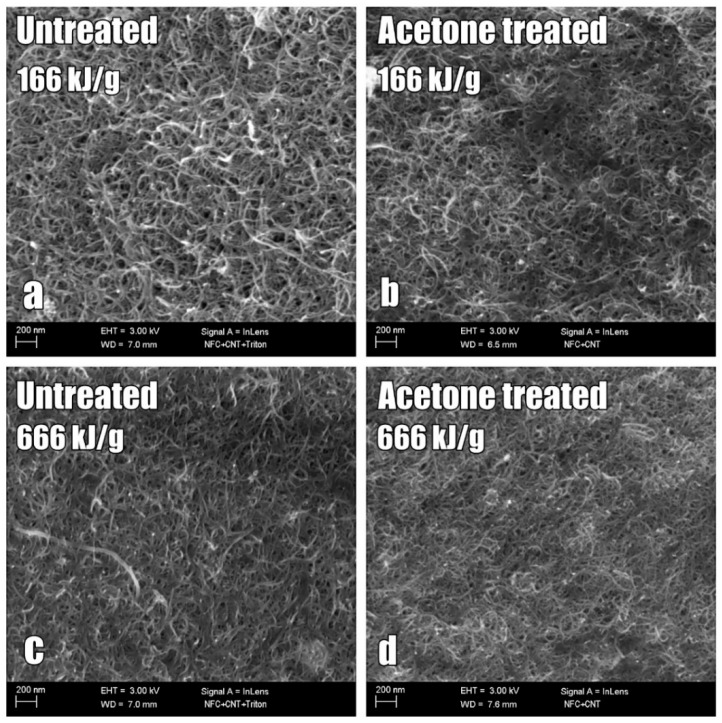
SEM imaging of NFC-CNT nanocomposite films surface (166 and 666 kJ/g) containing surfactant (**a**,**c**) and after removal of surfactant Triton X-100 by washing them in acetone (**b**,**d**).

The surface and cross-section of the films processed using surfactant Triton X-100 was studied with SEM. Images were taken with Zeiss ULTRAPlus scanning electron microscope (SEM). The effect of sonication as well as the removal of surfactant were studied with the SEM images shown in Figure 4. Two samples were specifically chosen for this inspection: 166 kJ/g and 666 kJ/g sonication energy films containing surfactant (Figure 4a,c) and after removal of surfactant Triton X-100 by washing them in acetone (Figure 4b,d).

In the top left (Figure 4a) image, some clusters of CNTs are present in the 166 kJ/g sonication energy sample, but not in the higher sonication energy 666 kJ/g sample. Both films have abundant amount of CNTs in the surface. Here, the 166 kJ/g film has a more porous structure than the 666 kJ/g film. In addition, the CNTs form a more consistent network in the 666 kJ/g film after washing the surfactant away (Figure 4d).

The SEM images of the sonicated samples in Figure 4 showed that there were clusters present in the 166 kJ/g sonicated film, while the higher sonicated energy sample did not have similar kinds of clusters. This indicates that, for lower sonication energies, non-dispersed particles remain in the films. This is not preferred, since the purpose is to achieve good dispersion of all the particles in the dispersion and in the films manufactured. This is an indication that the sonication process and amount of energy used affect the extent of the dispersion of particles.

**Figure 5:**
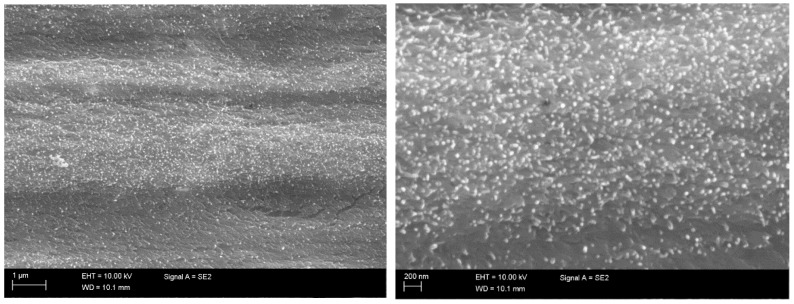
SEM imaging of the nanocomposite film (Triton X-100, 500 kJ/g) cross-section when embedded in epoxy: **Left** side: overall structure; **Right** side: magnification in the center of the film.

The through-thickness structure of the films was also generally studied using polished cross-sections of the films embedded in epoxy. The cross-section in Figure 5 shows the CNT ends (bright contrast spots) and their even distribution in the film (500 kJ/g), (Triton system) through the thickness. A slightly layered structure can be observed and concluded as a result of dispersion flow during the casting.

## 3. Discussion

Ultrasonication is a widely used process to manufacture aqueous CNT dispersions. However, how much it changes the properties of dispersed particles and the medium is often overlooked. It is known that sonication can, for example, generate hydrogen peroxide from water, degrade carbon nanotubes and ultimately destroy them. Therefore, it is important that the sonication process is optimized in terms of time and power. It also needs to be noted that the dispersion process is not linear, but follows an S-curve where temporal development of dispersion quality is related to quantity of un-dispersed solid.

Due to the re-agglomeration tendency of carbon nanotubes, it is necessary to use dispersion agents, i.e., surfactants, in manufacturing aqueous dispersions. If these dispersions are later used in conductive films, it is preferable to remove the surfactant to improve CNT network formation. Although carbon nanotubes are excellent conductors, CNT networks are not. This is due their high intertubular contact resistance. The contact points act essentially as a tunneling junction for electrons that is very sensitive to distance. The efficacy of surfactants is based on acting as a spacer between tubes, so any additional distance in a conductive network is detrimental to the conductivity itself.

## 4. Materials and Methods

Three-component systems containing nanocellulose, carbon nanotubes and surfactants are used in the strong, ecologically conscious nanocomposite films of this study. The CNTs add functionality to the nanocellulose matrix and the surfactant enables percolation network to build and maximize conductivity properties.

In this study, the nanocellulose (NFC) production was based on mechanical disintegration of bleached hardwood kraft pulp (BHKP). First, dried commercial BHKP produced from birch was soaked in water at approximately 1.7 wt. % concentration and dispersed using a high-shear Ystral dissolver for 10 min at 700 rpm. The chemical pulp suspension was predefined in a Masuko grinder (Supermasscolloider MKZA10-15J, Masuko Sangyo Co., Tokyo, Japan) at 1500 rpm and fluidized with six passes through a Microfluidizer (Microfluidics M-7115-30, Microfluidics Corp., Newton, MA, USA) using 1800 MPa pressure. The final material appearance of NCF was a viscous and translucent gel.

Multiwall carbon nanotubes (MWCNT, Nanocyl 7000, Nanocyl SA., Sambreville, Belgium) were purchased from Nanocyl Inc. and the product was used in the state it was in when received. This type of nanotubes is produced via catalytic chemical vapor deposition (CCVD). Concentration of CNTs was kept constant at 16 wt. % in the nanocomposite films, so the effects of sonication energy and surfactant type to the conductivity properties are more visible.

Three surfactants were chosen based on their ionic nature and standard: non-ionic Triton X-100 and Pluronic F-127, and anionic cetyl triammonium bromide (CTAB). Surfactants were purchased from Sigma-Aldrich (Merck KGaA, Darmstadt, Germany). The surfactants were diluted in deionized water to form solutions with variating dissolutions (1, 2.5, 10 wt. %).

### Preparation of NFC-CNT Aqueous Dispersion

**Figure 6:**
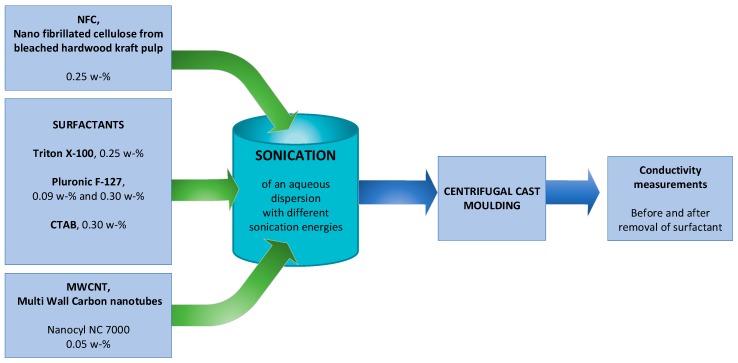
Preparation procedure of NFC-CNT dispersion and nanocomposite films.

The NFC and CNT were sonicated simultaneously and after sonication no centrifuge was used so that the preparation of aqueous dispersions could be achieved using a minimum amount of processing phases. NFC-CNT aqueous dispersions with a total volume of 80 mL were prepared. One set contained NFC (0.25 wt. %), CNTs (0.05 wt. %), deionized water and one of the selected surfactants (Triton X-100, 0.25 wt. %, Pluronic F-127, 0.09 wt. % and 0.3 wt. % and CTAB 0.30 wt. %). Details about preparation produce are showed as Figure 6.

The total dry mass for all the dispersions was 0.24 g. The sonication of the dispersion samples was performed with a tip horn (ø 12.7 mm) sonicator Q700 (QSonica LLC., Newton, CT, USA) in 100 mL glass beakers. The sonication amplitude of vibration (50%) was kept constant. The power output remained between 60 and 70 W for every sonication. The system included a water bath to keep samples cool during the sonication so that temperature would not rise above 30 °C. The water bath was cooled by circulating cooling glycerol through a chiller (PerkinElmer C6 Chiller, PerkinElmer Inc., Waltham, MA, USA). Samples were sonicated for four different amounts of energies per dry mass, respectively 166, 333, 500 and 666 kJ/g, which corresponded to energies of 40, 80, 120 and 160 kJ. Unsonicated samples manufactured using all three surfactants were not homogenous, and this is why film formation was unsuccessful and not analyzed.

## 5. Conclusions

The typical approach to the manufacturing of conductive cellulose-CNT films has been to increase CNT weight percentage without optimizing the dispersion procedure. In this study, NFC and multiwall carbon nanotubes (MWCNT) were used to prepare composite films using a minimum number of processing phases (e.g., no centrifugal dispersion or pressing of the film were used), materials and chemicals. The amount of CNTs was 0.05 wt. % in dispersion and 16 wt. % in the film after the evaporation of water in ratio to dry mass content of NFC and CNT. The effect of surfactant type (Triton X-100, Pluronic F-127 and CTAB) and sonication energy on the electrical conductivity of NFC-CNT nanocomposite films was investigated to identify optimal processing conditions for high conductivity of the nanocomposite. A conductivity of 5.36 S/cm was achieved by using Pluronic F-127 surfactant and 666 kJ/g of sonication energy. In addition, removal of the surfactant from film and its effect on the electrical conductivity was studied. A dramatic increase in conductivity values from approximately 3.0 S/cm to a value of 8.42 S/cm was observed for films manufactured with surfactant Triton X-100. Conductivity diagrams of the nanocomposite films show that sonication affects the electrical performance of the films. SEM images of sonicated samples showed that the films sonicated at 166 kJ/g have a more porous structure than the films sonicated at higher energy. The imaging also showed that the CNTs form a more consistent network with a combination of high sonication energy and surfactant removal. It can be concluded that the following parameters significantly affect the conductivity of NFC-CNT nanocomposite films:

(a) Surfactant type
(b) Surfactant concentration
(c) Sonication energy
(d) Removal of the used surfactant
(e) Film processing technique

To summarize, we manufactured nanocomposite films with exemplary conductivity in comparison to reported research and this was achieved by optimizing processing parameters and materials. Further research on the surfactant types and concentration can lead to better dispersion of the CNTs and therefore even higher conductivity.

## Figures and Tables

**Table 1:** Surfactants used in this study.

Product Name	Triton™ x-100	Pluronic^®^ F-127	CTAB
Type	Non-ionic	Non-ionic Polymeric	Cationic
Name	Octylphenol Ethoxylate	Poloxamer	Hexadecyltri-methylammonium bromide
Chemical Structure	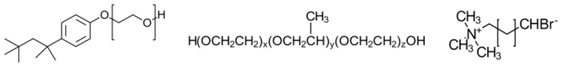		
Critical Micelle Concentration	0.2–0.9 mM (20–25) °C	950–1000 ppm (25 °C)	0.92 mM (20–25) °C
HLB value	13.5	22	10
